# The Sensitivity of Massively Parallel Sequencing for Detecting Candidate Infectious Agents Associated with Human Tissue

**DOI:** 10.1371/journal.pone.0019838

**Published:** 2011-05-13

**Authors:** Richard A. Moore, René L. Warren, J. Douglas Freeman, Julia A. Gustavsen, Caroline Chénard, Jan M. Friedman, Curtis A. Suttle, Yongjun Zhao, Robert A. Holt

**Affiliations:** 1 Genome Sciences Centre, BC Cancer Agency, Vancouver, British Columbia, Canada; 2 Department of Earth and Ocean Sciences, University of British Columbia, Vancouver, British Columbia, Canada; 3 Department of Medical Genetics, University of British Columbia, Child and Family Research Institute, Vancouver, British Columbia, Canada; 4 Departments of Earth and Ocean Sciences, Botany, and Microbiology and Immunology, University of British Columbia, Vancouver, British Columbia, Canada; Georgia Institute of Technology, United States of America

## Abstract

Massively parallel sequencing technology now provides the opportunity to sample the transcriptome of a given tissue comprehensively. Transcripts at only a few copies per cell are readily detectable, allowing the discovery of low abundance viral and bacterial transcripts in human tissue samples. Here we describe an approach for mining large sequence data sets for the presence of microbial sequences. Further, we demonstrate the sensitivity of this approach by sequencing human RNA-seq libraries spiked with decreasing amounts of an RNA-virus. At a modest depth of sequencing, viral transcripts can be detected at frequencies less than 1 in 1,000,000. With current sequencing platforms approaching outputs of one billion reads per run, this is a highly sensitive method for detecting putative infectious agents associated with human tissues.

## Introduction

Infectious agents (IAs) are a common cause of acute and chronic human diseases. For example, it is estimated that approximately 20% of all human cancers are caused by an infectious agent [1], [2]. Many infectious causes of human disease have likely remained undiscovered because of limitations of traditional, culture-based identification methods. Metagenomic sequencing has high sensitivity and offers the possibility of identifying new IAs, should they exist. Examples of conditions with potential associations to IAs include autoimmune diseases such as multiple sclerosis and inflammatory bowel disease and malignancies such as oropharangeal cancer, colorectal cancer and childhood leukaemia/lymphomas.

Previous studies have focused on a targeted approach using RT-PCR or conventional detection methods to confirm disease associations with known IAs. The expansion in sequencing capacity enabled by massively parallel sequencing platforms allows for the first time complete and unbiased surveys of the human genome, transcriptome and microbiome. Several recent studies have demonstrated the utility of this approach for detecting IAs; examples include the discovery of a novel polyomavirus in Merkel Cell carcinoma [3], and identification of a previously unknown arenavirus associated with the deaths of three human organ transplant recipients [4].

Although many investigators are using metagenomic sequencing for the purpose of detecting novel IAs, a rigorous empirical study of IA detection level in human tissues is missing. This kind of experiment is necessary to determine sequencing depth and to establish precisely the amount of IA expression that can be reliably detected. Such an experiment also provides a proof of principle for the methodology, by illustrating that known IAs can be detected if present at a certain level of abundance. This information will be useful for any group attempting IA detection using sequence data generated on a massively parallel platform. The data presented here establishes a detection limit using RNA sequencing data from a human colorectal tumour biopsy tissue spiked with viral RNA.

**Figure 1:**
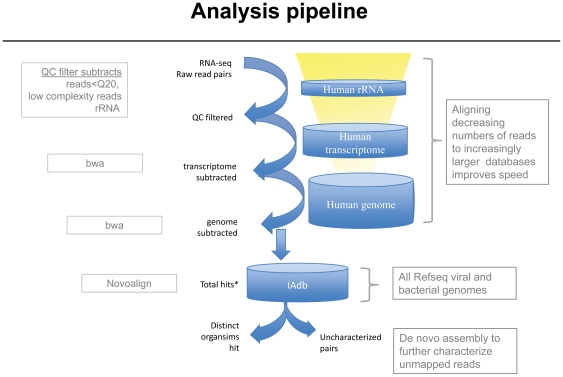
Flow chart of subtraction methodology. Paired end reads from a human sequence library are first filtered to remove low quality reads; <Q20 average, reads with low complexity (> = 20 nt homopolymers), and reads comprised of artifactual adapter or primer sequences. Then, using BWA [9], read pairs are aligned to databases of human ribosomal sequences, transcript sequences, and genomic sequences. Remaining reads are then aligned sequentially to the genome, transcriptome and human rRNA using BWA [9]. Reads that remain unaligned after comparison to the various human sequence databases are then aligned to a custom database (IAdb) of all known viral and bacterial complete genome sequences, using Novoalign (http://www.novocraft.com), with the requirement of correct pairing logic. Although not considered here, remaining reads can be characterized further by de novo assembly.

We have implemented a bioinformatics strategy (Fig. 1) for the detection of putative pathogen sequences from large data sets of short sequence reads, such as those generated by the Illumina GAIIx and HiSeq. Mate-pair sequences are aligned sequentially against non repeat-masked, same-species rRNA, cDNA and genome reference sequences, and any reads aligning above user-defined thresholds are subtracted. The reads that remain unaligned to any of the host species sequences are then used to interrogate a collection of all known bacterial and viral genome sequences. The identity of microorganisms in the original tissue specimen is then inferred from sequence matches. Although in principle either genome or transcriptome sequence can be interrogated, we prefer to analyze transcriptomes by this approach. Whole transcriptome shotgun sequencing (WTSS or RNAseq) provides in depth sampling at reduced cost. A comprehensive transcriptome analysis can be performed with approximately 1/10^th^ the number of bases (10 Gb) vs. the 100 Gb required for whole genome sequencing. Transcripts at only a few copies per cell are readily detectable, allowing the discovery of even low abundance viral and bacterial transcripts. Transcriptome analysis also has the advantage of only reporting actively expressed transcripts rather than just the presence of a particular sequence. In the case of IA gene expression, this implies a much greater likelihood of disease involvement, especially if expression levels can be compared to corresponding normal tissue from the same individual.

## Results

The capability to detect candidate infectious agents in a human sequencing library was evaluated using spiked-in RNA virus and computational host sequence subtraction. In order to determine sensitivity experimentally, a known IA was required as a template. RNA isolated from a single stranded RNA virus was chosen to enable testing of the entire procedure, including cDNA synthesis and sequence library construction. A non-human virus was selected to ensure all detected reads would be derived from the spiked material and not from native expression. The virus chosen was Heterosigma akashiwo RNA virus (HaRNAV), which infects the toxic bloom-forming unicellular alga, *Heterosigma akashiwo* (*Raphidophyceae*) [5]. This positive-sense single stranded RNA virus (Picornavirales) has a fully sequenced 8.6 kbp genome [6] (GenBank: AY337486.1) and as such was a part of our in-house infectious agent database. HaRNAV has a GC content of 46%.

HaRNAV RNA was quantified by Nanodrop® and added over 4 log dilutions to aliquots of total RNA extracted from a snap-frozen human biopsy sample (details in methods section). Four libraries were prepared, each from 2 µg of total human RNA (RNA integrity number 9.1), to which 0.2 ng, 20 pg, 2 pg and 0.2 pg, respectively, of viral RNA was added. The viral samples were prepared as 10-fold serial dilutions and HaRNAV would be expected to be recovered 1 in 10,000, 1 in 100,000, 1 in 1 million and 1 in 10 million reads, respectively. DNA from a marine cyanophage (SPWM3) was added at the opposite ratios as HaRNAV to the cDNA preparation, also over 4 log dilutions. This spike in at the cDNA level served as a detection control for the steps following cDNA generation and was included as a comparator to the RNA spike in. 100 µl of a 10 pM concentration of each of the resulting libraries were run on an Illumina GAIIx sequencer. The resulting data from each library were of high and equivalent quality, with the percentage of bases >Q30 ranging from 87.1% to 88.8% and a post purity filtered rate of 80% to 84%, number of reads are noted in Table 1 and 2. Further analysis was blinded to the identity and concentrations of the spiked viral sample, allowing an unbiased assessment.

**Table 1:** Summary of hits to IAdb.

Library	HaRNAV		Bacteriophage	
	Raw read pairs	Rank #	Raw read pairs	Rank #
1	547	4	2	81
2	37	17	4	49
3	6	77	132	19
4	1	294	2,078	7

Rank # is the order of the detected IA characterized by decreasing pair counts (i.e. the genome to which most read pairs align ranks #1).

**Table 2:** Sequence summary and detection of viral DNA.

Library	Human RNA (µg)	Viral RNA (pg)	Dilution factor	Read pairs	Total HaRNAV read pairs expected	HaRNAV			Bacteriophage		
						Read pairs	ppm*	Expected ppm	Read pairs	ppm	Expected ppm
1	2	200	1∶10,000	20,352,714	2,035	618	30.36	100	2	0.10	0.1
2	2	20	1∶100,000	23,334,389	233	31	1.33	10	3	0.13	1
3	2	2	1∶1,000,000	22,504,865	23	6	0.27	1	137	6.09	10
4	2	0.2	1∶10,000,000	22,224,735	2	1	0.04	0.1	2,162	97.28	100

*ppm: pairs per million.

RNA-seq data were quality filtered to remove reads having either an average base quality below phred 20 [7], [8] and/or more than 20 consecutive homopolymeric bases were subtracted from the original data. Artifactual reads comprised of primer or adapter sequences were also removed at this stage. Quality filtered read pairs were then aligned with bwa (version 0.5.4 [sample -o 1000, default options]) [9], sequentially against well-annotated human rRNA, cDNA and genome reference sequences (Ensembl.org). Bwa is a short, high stringency read aligner with fast execution, ideal for processing short (<200 bp) high quality read pairs that have high sequence similarity (<3% mismatch) to the reference human sequences. In the present study, although excess human rRNA was removed before cDNA synthesis by column hybridization (see methods), for the four libraries residual rRNA still comprised 19.9+/−1.21% of the sequence data. The total number of read pairs removed after QC (including rRNA, low complexity and low quality average<Q20) was 9.8+/−0.9 million (mean +/− SD).

Read pairs that remained unaligned to any of the human sequence databases were used to interrogate a custom database that contains all completely sequenced bacterial and viral genomes from Genbank. This in-house database, which we call IAdb is frequently updated to remain current. The version of IAdb used for this manuscript was composed of 27,699 sequences totaling 3.9 Gbp, including 23,163 sequences from 1,016 bacterial genomes. (ftp://ftp.ncbi.nlm.nih.gov/genomes/Bacteria) and 3,536 (72.6 Mbp) viral refseq sequences (ftp://ftp.ncbi.nih.gov/refseq/release/viral). It is publicly available for download at ftp://ftp.bcgsc.ca/supplementary/IADB/. For alignments to the IAdb, Novoalign (http://www.novocraft.com/) was used (version 2.05.20 [-o SAM -r A -R 0, default options] (http://www.novocraft.com/) which reports all locations and genomes mapped equally well by a given read pair. Novoalign is a more permissive but slower short read aligner than bwa that takes into account quality scores. These features make it more suitable than bwa for identifying metagenomic sequences, which may have limited similarity to the reference sequences in IAdb. Counts of read pairs matching each Genbank accession in our IAdb were obtained. However, for the purpose of this study, we only tallied read pairs that mapped unambiguously, that is, where both members of the mate pair matched the same accession and with the correct pairing distance and orientation.

In our overall approach sequential subtraction of host sequences serves two purposes 1) it speeds up the final alignment to IAdb, by weeding out decreasing number of pairs aligned against increasingly larger sequence databases and 2) it helps remove the background noise. For the present study alignments were run on a single 3 GHz 8 CPU Intel(R) Xeon(R) 64-bit 61 GB RAM computer running CentOS release 5.4.

Table 1 demonstrates detection of the spiked HaRNAV genetic material using the blinded data. For library 1, which contained the highest concentration of viral RNA, HaRNAV was one of the most highly expressed agents, and was still in the top 100 for pair abundance in subsequent libraries 2 and 3. As these 4 libraries were technical replicates made from the same total RNA preparation all other IA levels were similar across all libraries. This simplified the detection of the spiked sample especially at the lower limit. Reads were normalized to the library with the fewest number of reads after the removal of those reads that aligned to rRNA (library 1). A summary of the observed and expected numbers of reads is shown in table 2.

These data illustrate robust recovery of both the HaRNAV RNA and the phage DNA in terms of positive hits with the expected pairs per million being proportional to those observed (Pearson correlation >0.99). However the number of RNA viral read pairs in each library was about 1/3 of that expected, based on the amount of material spiked into the library. This discrepancy may be due to the difficulty of accurate quantification of ssRNA viruses coupled with a preferential capture of smaller RNA products in the library preparation. We have demonstrated with the phage spike in that recovery was close to expected after the cDNA stage again indicating that the bias was at an early stage of library preparation. From the simulated data presented in table 3 we know that HaRNAV derived read pairs are not lost in the bioinformatic pipeline to any significant extent. Possible sources of data attrition also include disproportional filtering, for example, of homopolymeric genomic regions. Likewise, low quality bases (threshold set as a minimum average of phred 20 for this study) could cause pairs to evade detection.

**Table 3:** Effect of read length and base error on HaRNAV and Herpesvirus 4 detection.

Conditions			HaRNAV	Herpesvirus-4
Read Length (nt)	Error on genome	Number of simulated pairs tested	Mean #pairs detected +− std.dev.	Mean #pairs detected +− std.dev.
76	0%	1000	1000.0+−0.0	1000.0+−0.0
76	3%	1000	1000.0+−0.0	999.3+−1.3
76	5%	1000	979.0+−12.0	973.0+−4.0
36	3%	1000	1000.0+−0.0	1000.0+−0.0
50	3%	1000	1000.0+−0.0	999.3+−1.3
100	3%	1000	985.7+−2.1	989.7+−2.5

**Figure 2:**
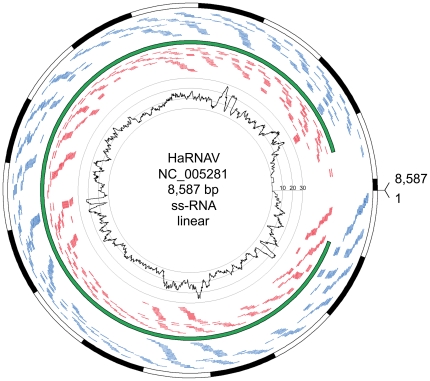
Circos [16]plot detailing HaRNAV sequence recovery. The red and blue lines represent reads aligning on the minus and plus strand, respectively. The Heterosigma akashiwo RNA virus has an 8,587 bp ss-RNA linear genome with a single CDS, shown in green on the circos plot. The read depth of coverage is shown in the centre of the plot. The genome is depicted by alternating black-white arcs of 500 bp in size.

Nevertheless, minute amounts of viral RNA were detected, corresponding to an average of less than one copy per cell (for further details see methods). Using all read pairs that aligned to HaRNAV in RAM01, we observe 97.65% (8385/8587) coverage of the viral genome and a largely uniform average depth of coverage of 9.7 fold, (Fig. 2).

### IA detection pipeline accuracy

To evaluate further the performance of the bioinformatics pipeline we randomly generated, in triplicate experiments, 1, 10, 100 and 1000 paired reads from the RNA virus HaRNAV (NC_005281.1) and DNA virus Human herpesvirus 4 (NC_009334.1) genomes, respectively and ran these faux read pairs through the IA detection pipeline. The simulation tested the effect of read lengths of 36, 50, 76 and 100 nt and error rates by simulating HaRNAV and herpesvirus genomes having up to 5% simulated error.

We observe that mapping is minimally impacted by errors, consistent with the permissive limit on the mapping accuracy of Novoalign, the aligner used to search IAdb (Table 3). Because errors were simulated at random on viral entries, they are not evenly distributed and the longer the read, the higher the chance of incurring more than the allowed number of mismatched bases during alignment. Thus, for an aligner such as Novoalign that uses a threshold of a fixed number of mismatches, rather than accept an error frequency, longer reads may actually impair the detection of non-identical, but highly related species. However, Novoalign's permissive nature is of utility here, in that it allows mapping of reads with lower stringency than bwa, which enables the identification of putative IA agents that are not identical to known genomes. Until public sequence repositories become many orders of magnitude more comprehensive and allow accurate species identification, using a permissive aligner in conjunction with 75–100 nt read pairs is a just proposition for IA detection

The mapping accuracy of bwa (<3%) could be limiting in identifying retroviruses whose identity is close to that of human endogenous retroviral (HERV) sequences, for instance, but unrestrictive if sequences are sufficiently (>3%) divergent. To test this hypothesis, we challenged the IA detection pipeline in identifying and subtracting HERV-derived read pairs while detecting HERV-like viruses-derived pairs. HERVs are remnants of ancient retroviral genomes integrated in human germline DNA, some of which are transcribed. A HERV-K sequence (DQ821442.1) encoding a reverse transcriptase (*pol)* and its closest known viral genome homologue (FJ979639.1) deposited in the Genbank NT database, a region of the Simian retrovirus 4 (SRV4) strain having 60% sequence identity with HERV-K, were chosen for the simulation. When reads were simulated with no errors, none of the HERV-K pairs passed the pipeline beyond human transcriptome alignments and all the SRV4 were recovered. When we applied a 5% simulated mutation rate to both sequences and simulated 76 nt paired reads, we observe that in average 76+/−35.4 read pairs escaped the transcriptome filtration for HERV-K when testing 1000 pairs, due to corresponding read pairs that accrued more than bwa's maximum allowable limit of 3% errors. The number of unfiltered pairs is low despite a substantial (5%) mismatch rate, because when at least one read within a pair still maps to a human sequence target with more than 97% accuracy the pair is removed. This is in fact the case for 92.4% of pairs simulated from an HERV-K sequence having 5% randomly distributed errors. In contrast, none of the SRV4 faux pairs are subtracted, since the sequence is sufficiently divergent from human sequences, including HERVs. The pipeline is thus conservative in that it is designed to stringently remove as much of the host “noise” as possible before the final, more permissive mapping to viral/bacterial genomes.

To address the issue of detecting unknown agents, we have ran the IA detection pipeline on simulated HaRNAV and herpesvirus read pairs, as described above, but using a modified IAdb depleted of the HaRNAV (NC_005281.1) and herpesvirus 4 (NC_009334.1) genome sequence entries, respectively. Whereas we do not detect any HaRNAV reads in the former experiment because of lack of similar entries in IAdb, we detect herpesvirus pairs, due to the overrepresentation of highly similar herpesvirus species in public sequence data repositories (Table 4). This highlights that success in identifying a putative IA with this set of tools is dependent on how wide-ranging the sequence database is. Different approaches could be designed to help make sense of reads unmapped from IAdb, reads with the potential to characterize poorly annotated agents not represented in the highly curated IAdb. For example, unmapped pairs could be assembled *de novo* and the resulting contigs aligned onto a more comprehensive, but fragmented, sequence database such as Genbank NT.

**Table 4:** Effect of IAdb entry removal on viral sequence detection.

Conditions			HaRNAV	Herpesvirus 4
Read Length (nt)	Error on genome	Number of simulated pairs tested	Mean #pairs detected* +− std.dev.	Mean #pairs detected +− std.dev.
76	3%	1	0.0+−0.0	1.0+−0.0**
		10	0.0+−0.0	8.7+−1.5
		100	0.0+−0.0	90.3+−5.0
		1000	0.0+−0.0	978.3+−3.5

*Upon depletion of HaRNAV and Herpesvirus 4, respectively and listing pairs that hit other entries. ** Pairs map unambiguously to AY961628.3, Human herpesvirus 4 strain GD1 and/or NC_007605.1, Human herpesvirus 4 type 1. Missing pairs were neither subtracted at the human sequence screening phase, nor mapped to known viral/bacterial entries and may characterize uniquely the human herpesvirus 4 genome, accession NC_009334.1.

## Discussion

There is a worldwide effort towards sequencing the human microbiome [10]. This work may benefit from the present study, which clearly demonstrates the feasibility of using massively parallel sequencing technology to detect an RNA virus present at very low levels in human tissue. The detection level we established can be used to estimate the depth needed to detect a candidate IA, number of IA genomes per cell, or depth required to assemble full IA transcripts. This will help inform study design by, for example, guiding decisions on amounts of DNA and tissue required per sample and number of samples needed for the study.

In contrast to previous metagenomic IA detection approaches that used pyrosequencing [3], [11] the Illumina platform allows an order of magnitude greater depth in sequencing reads that is reflected in a very low detection level. Proof of principle using this platform has been illustrated by the detection of HPV E6-E7 expression in HeLa [12], clearly demonstrating the IA expression can be captured. The use of a comprehensive infectious agent database allows detection without the need for sequence assembly, however, a limitation of this technique is that that totally novel IA candidates could be missed if their expressed genes are not closely related to any known agent. In this regard, as useful approach may be pre-assembly of host-filtered reads to generate contigs that are longer and more easily characterized than individual reads

Finally, it is well understood that the discovery of an IA association with disease is only the first stage in determining causality. Further validation studies to determine statistical significance in larger sets are needed as well as independent evidence of pathogenicity.

## Methods

HaRNAV was amplified using the Raphidophyte *Heterosigma akashiwo* (NEPCC 522). Cultures (400 mL) were grown in 1 L Erlenmeyer flasks (Nalgene) in a 14∶10 light:dark cycle (ca. 250 µmol quanta m^−2^ s−^1^ photosynthetically active radiation) at 19°C, and the viruses amplified as outlined in Tai *et al*. (2003).

HaRNAV was concentrated by ultracentifugation of 78 mL of lysate at 108 000 x g for 5 h, and the pelleted virus resuspended in 75 µl of supernatant. The concentrated virus was brought to a final concentration of 5 mM MgCl_2_ and incubated with 0.1 U/µL of RNase A (Invitrogen) and 1 U/µL of Amplification Grade DNAse 1 (Invitrogen) for 3 h at room temperature. The viruses were extracted with Trizol (Invitrogen) as per manufacturer's instructions and resuspended in 20 µL RNaseAway (Invitrogen).

Bacteriophage S-PWM3 was isolated from the Gulf of Mexico using the cyanobacterium *Synechococcus* strain DC2 (WH7803) as the host [13], grown at 23°C in artificial seawater under 10–20 µmol quanta m^−2^ s^−1^. The viruses were amplified by adding the bacteriophage to 500 mL of exponentially growing *Synechococcus* at a virus to host ratio of 1 [14]. After complete lysis, the lysate was filtered through a 0.22-µm pore-size GVWP filter (Millipore) to remove cellular debris. The filtrate was centrifuged for 5 h at 108,000 x g and the pelleted viruses resuspended in 200 µL of sterile artificial seawater. Phage DNA was extracted using the viral QIAamp MinElute Virus Spin Kit (Qiagen, Mississauga, Canada) according to the manufacturer's instructions.

Sample tissue, a colorectal tumour biopsy, was acquired from the BCCA Tumour Tissue Respository (TTR) as a frozen biopsy. A maximum of 30 mg of tissue was homogenized in 600 µl RLT buffer (Qiagen) and passed 5 times through a syringe fitted with a 20 G needle. RNA was purified using the RNeasy Plus Mini Kit (Qiagen) following the manufacturer's instructions. Genomic DNA contamination was reduced using an on-column DNase I treatment according to the kit protocol.

Total RNA was quantified using Qbit. RNA quality was assessed using Agilent Bioanalyzer 2000 RNA nanochip. 2 µg of total RNA was used in each library construction. Briefly, ribosomal RNAs (rRNAs) were depleted from total RNA by following the protocol of RiboMinus Eukaryote Kit for RNA-Seq (Invitrogen, Carlsbad, CA). Libraries were then constructed according to BCCA Genome Sciences Center RNA-Seq paired-end library construction protocol with an approximate 200 bp inset size [15].

Libraries were sequenced on the Illumina GAIIx system using a paired end strategy at a read length of 75 bases.

Alignments were performed using BWA v0.5.4 [sample -o 1000] [9] and Novoalign v2.05.20 [-o SAM -r A -R 0] (http://www.novocraft.com).

In order to determine the detection level, the size of the viral RNA genome (4.4 Kb) was used to calculate the mass of the genome, producing an estimate that 1 pg of viral RNA constitutes approximately 150,000 copies. In order to estimate the number of viral genomes per cell, we assumed that an average cell has 20 pg of total RNA. Therefore 2 µg of total RNA, as used in our library preparation, equates to approximately 100,000 cells.
